# Dietary habits in adult Japanese patients with vitiligo

**DOI:** 10.1111/1346-8138.17163

**Published:** 2024-02-29

**Authors:** Risa Hamada, Yoko Funasaka, Hidehisa Saeki, Naotaka Serizawa, Teppei Hagino, Yumiko Yano, Hiroshi Mitsui, Naoko Kanda

**Affiliations:** ^1^ Department of Dermatology Nippon Medical School Tokyo Japan; ^2^ Department of Dermatology Nippon Medical School Chiba Hokusoh Hospital Inzai Japan; ^3^ Tokyo Teishin Hospital Tokyo Japan

**Keywords:** age, body mass index, dietary habit, male, vitiligo

## Abstract

Vitiligo is an autoimmune skin disease with acquired depigmentation. Dietary habits may modulate the pathogenesis of vitiligo. We evaluated dietary habits in adult Japanese patients with nonsegmental vitiligo, and compared their results with those of age‐ and sex‐matched controls. We also examined the relationship between dietary habits and Vitiligo Area Scoring Index (VASI), or vitiligo on different anatomical sites. The intakes of energy, nutrients, and foods in the participants were analyzed using a brief‐type self‐administered diet history questionnaire. Patients with vitiligo showed higher body mass index (BMI) and lower intakes of manganese, vitamin D, pulses, and confection, compared with controls. Multivariate logistic regression analysis showed that vitiligo was associated with high BMI. VASI was higher in males than in females, and negatively correlated with age or intakes of potatoes and vegetables other than green/yellow vegetables. Linear multivariate regression analysis showed that high VASI was associated with younger age. Multivariate logistic regression analysis showed that moderate to severe vitiligo (VASI ≥ 4.25) was associated with male sex and longer disease duration. Multivariate logistic regression analyses showed the following association with vitiligo on respective anatomical sites: high intake of eggs and dairy products and high VASI on the head or neck, high intake of oils and fats and high VASI on the trunk, high intake of cereals and high VASI on the upper limbs, male sex and high VASI on the lower limbs, and high BMI and high VASI on the hands or feet. In conclusion, the control of obesity might have prophylactic or therapeutic effects on vitiligo.

## 1 INTRODUCTION

Vitiligo is an autoimmune skin disorder characterized by depigmented patches due to the dysfunctional melanocytes attacked by immune cells.^1^ The pathogenesis of vitiligo is multifactorial, related to melanocyte dysfunction by genetic susceptibility, dysregulated oxidative stress, inflammation, and autoimmune responses.^2^ In melanocytes, certain stimuli such as UV induce melanogenesis, the production of melanin in melanosomes. In the process of melanogenesis, reactive oxygen species (ROS) are produced. Under normal conditions, antioxidative nuclear erythroid 2–related factor 2 (Nrf2) functions to promote the autophagy or the expression of antioxidative enzymes. However, in vitiligo, such resistance to oxidative stress is defected,^3^, ^4^ and excessive ROS accumulate, leading to apoptosis, necrosis, or ferroptosis of melanocytes. The oxidative stress induces the production of 70‐kDa heat shock protein (Hsp70) or inflammatory cytokines such as interleukin (IL)15, tumor necrosis factor‐α (TNF‐α), IL‐6, or chemokines CXCL9/10/16 from stressed keratinocytes or melanocytes. These cytokines/chemokines or Hsp70 promote the activation and recruitment of dendritic cells (DCs), CD8+ cytotoxic T lymphocytes (CTLs), interferon‐γ (IFN‐γ)–producing T helper 1 (Th1) cells, or IL‐17–producing Th17 cells while downregulate regulatory T cells (Tregs). The melanocytes that have died release autoantigens, such as gp100, which are carried by DCs and presented to autoreactive CTLs. Autoreactive CTLs release perforin and granzyme B, inducing apoptosis of melanocytes,^2^ and IFN‐γ and TNF‐α, inducing the detachment of melanocytes from keratinocytes by reducing E‐cadherin expression.^1^ The CD8+CD103+CD69+CD49a+ resident memory T cells (T_RM_) and keratinocyte‐derived IL‐15, a cytokine sustaining T_RM_, contribute to the perpetuation and recurrence of vitiligo.^2^, ^5^, ^6^

Genetic factors, such as HLA‐A*02:01,^7^ and environmental factors, such as UV or pollutants, are involved in the pathogenesis of vitiligo. Diet is one such environmental factor. The deficiency or excess of certain nutrients might alter the immune responses or redox status of melanocyte‐surrounding microenvironments. It is reported that serum 25‐hydroxyvitamin D levels were lower in patients with vitiligo than in controls,^8^, ^9^ indicating the relationship between vitamin D deficiency and vitiligo. It is reported that oral intake of α‐lipoic acid, vitamin C, vitamin E, and polyunsaturated fatty acids improves the treatment efficacy of narrowband UV‐B in patients with vitiligo, together with increased catalase activity in peripheral blood mononuclear cells.^10^ The results indicate that nutrients acting as antioxidants may have supplementary therapeutic effects on vitiligo. The nutritional status is altered by dietary habits; however, the dietary habits in patients with vitiligo have not been precisely examined.

Herein, we investigated the dietary habits of adult Japanese patients with vitiligo and compared the results with those of age‐ and sex‐matched healthy controls, using a brief‐type self‐administered diet history questionnaire (BDHQ).^11^ We also examined the relationship between dietary habits and disease severity or the presence of vitiligo on different anatomical sites.

## 2 METHODS

### 2.1 Study population

This study was performed in accordance with the Declaration of Helsinki (2004) and was approved by the ethical committee of the affiliation. The purpose of the protocol was explained to the patients and controls, and written informed consent was obtained. Sixty Japanese patients clinically diagnosed as having nonsegmental vitiligo (31 males and 29 females) participated in this study. They visited the participating facilities during June 2022 to December 2023 and agreed to the study. At the time of dietary assessment, patients were treated as follows: 32 patients with topical delgocitinib, three patients with topical corticosteroids, two patients with topical maxacalcitol, two patients with topical tacrolimus; systemically, 16 patients with combination of ascorbic acid, tocopherol acetate, and l‐cysteine, one patient with upadacitinib, one patient with baricitinib, 13 patients with ritlecitinib, 19 patients with excimer light, and two patients with narrowband UV‐B. The association of autoimmune diseases was as follows: two patients with primary biliary cholangitis, two patients with Graves' disease, two patients with Hashimoto's thyroiditis, two patients with alopecia areata, one patient with rheumatoid arthritis, one patient with Sjögren syndrome, one patient with multiple sclerosis, and one patient with psoriatic arthritis. The association of the other diseases was as follows: one patient with sarcoidosis, four patients with hypertension, six patients with dyslipidemia, two patients with hyperuricemia, two patients with diabetes, one patient with atopic dermatitis, one patient with keloid, one patient with chronic liver disorder, and one patient with thoracic outlet syndrome. At the time of dietary assessment, severity of disease was assessed with the Vitiligo Area Scoring Index (VASI) as described.^12^ The patients were divided into two subgroups according to the presence or absence of vitiligo on the head or neck, trunk, upper limbs, lower limbs, or hands or feet, respectively (Table 1). The patients were also divided into mild (VASI < 4.25, *n* = 30) and moderate to severe (VASI ≥ 4.25, *n* = 30) vitiligo subgroups, by the median of the VASI.

**TABLE 1:** Demographic characteristics of controls and patients with vitiligo and their intakes of nutrients and foods.

	Controls (*n* = 60)	Patients with vitiligo (*n* = 60)	*p* Value
Sex			
Male	31	31	1^e^
Female	29	29	
Age (years)^a^	53.7 ± 15.3	53.8 ± 15.4	0.734
Disease duration (years)^b^	NA	7.5 (3.0–13.25)	NA
BMI (kg/m^2^)^b^	20.65 (19.375–24.45)	22.9 (21.375–25.675)	0.0107*
VASI^b^	NA	4.25 (0.61875–13.5)	NA
Vitiligo on the head or neck^c^	NA	48 (80)	NA
Vitiligo on the trunk^c^	NA	34 (56.7)	NA
Vitiligo on the upper limbs^c^	NA	34 (56.7)	NA
Vitiligo on the lower limbs^c^	NA	31 (51.7)	NA
Vitiligo on the hands or feet^c^	NA	32 (53.3)	NA
Energy intake (kcal/day)^b^	1924 (1543–2174)	1700 (1392–2099)	0.136
Nutrients			
Animal protein (% energy)^b^	8.225 (6.823–10.130)	7.855 (6.653–10.195)	0.814
Plant protein (% energy)^b^	6.306 ± 1.006	6.095 ± 1.335	0.383
Animal fat (% energy)^a^	13.567 ± 4.413	13.566 ± 4.544	0.999
Plant fat (% energy)^a^	14.588 ± 3.032	13.593 ± 4.108	0.151
Carbohydrate (% energy)^a^	50.075 ± 7.748	49.385 ± 8.474	0.653
Sodium (mg/kcal)^b^	2.145 (1.908–2.580)	2.300 (1.958–2.630)	0.536
Potassium (mg/kcal)^b^	1.375 (1.108–1.620)	1.225 (1.018–1.538)	0.141
Calcium (μg/kcal)^b^	279.38 (233.42–348.52)	244.85 (199.84–360.13)	0.968
Magnesium (μg/kcal)^b^	130.92 (115.69–154.93)	125.32 (112.08–154.56)	0.367
Phosphorus (μg/kcal)^b^	550.6 (472.0–671.2)	546.7 (459.5–624.0)	0.651
Iron (μg/kcal)^a^	4.276 ± 1.055	4.216 ± 1.212	0.76
Zinc (μg/kcal)^b^	4.365 (3.803–4.725)	4.375 (3.793–4.815)	0.953
Copper (μg/kcal)^a^	0.5873 ± 0.0975	0.5912 ± 0.1271	0.843
Manganese (μg/kcal)^b^	1.670 (1.435–1.9675)	1.390 (1.2175–1.865)	0.03998*
Retinol (μg/kcal)^b^	0.210 (0.150–0.363)	0.210 (0.138–0.370)	0.792
β‐Carotene (μg/kcal)^b^	1.715 (1.133–2.708)	1.520 (0.933–2.363)	0.162
Vitamin A (μg RAE/kcal)^b^ ^,^ ^d^	0.395 (0.280–0.583)	0.400 (0.260–0.545)	0.419
Vitamin D (ng/kcal)^b^	5.325 (3.825–8.205)	4.550 (3.595–6.9825)	1.76 × 10^−11^ **
α‐Tocopherol (μg/kcal)^b^	4.0915 ± 0.9079	3.8555 ± 1.1356	0.226
Vitamin K (μg/kcal)^b^	0.145 (0.108–0.21)	0.165 (0.108–0.220)	0.854
Vitamin B1 (μg/kcal)^a^	0.4315 ± 0.1062	0.2055 ± 0.4110	0.285
Vitamin B2 (μg/kcal)^a^	0.7363 ± 0.1729	0.7208 ± 0.2137	0.661
Niacin (μg/kcal)^a^	9.315 (8.358–11.070)	8.760 (7.750–10.603)	0.144
Vitamin B6 (μg/kcal)^b^	0.675 (0.580–0.825)	0.625 (0.558–0.743)	0.211
Vitamin B12 (ng/kcal)^b^	4.10 (3.008–5.825)	3.845 (2.790–5.125)	0.988
Folic acid (μg/kcal)^b^	0.180 (0.150–0.230)	0.170 (0.128–0.220)	0.126
Vitamin C (μg/kcal)^b^	56.365 (44.453–75.178)	47.115 (33.325–64.013)	0.119
SFA (% energy)^a^	7.884 ± 2.216	7.534 ± 2.308	0.437
MUFA (% energy)^a^	10.140 ± 2.152	9.684 ± 2.390	0.334
*n*‐3PUFA (% energy)^b^	1.225 (1.028–1.430)	1.200 (1.028–1.403)	0.64
*n*‐6PUFA (% energy)^a^	5.238 ± 1.2263	5.242 ± 1.396	0.993
Cholesterol (μg/kcal)^b^	205.49 (140.09–258.66)	189.47 (147.13–281.95)	0.828
Alcohol (% energy)^b^	3.255 (0–7.835)	2.800 (0–12.103)	0.351
Foods			
Cereals (mg/kcal)^b^	186.3 (159.6–225.5)	183.2 (152.1–249.7)	0.429
Potatoes (mg/kcal)^b^	17.83 (9.376–29.55)	13.79 (8.95–26.53)	0.155
Pulses (mg/kcal)^b^	2.054 (1.219–3.576)	1.777 (0.970–2.562)	3.21 × 10^−11^ **
Green and yellow vegetables (mg/kcal)^b^	59.820 (36.054–77.5)	56.279 (28.633–71.327)	0.732
Other vegetables (mg/kcal)^b^	79.512 (53.876–102.479)	67.100 (43.211–87.545)	0.0897
Fruit (mg/kcal)^b^	46.799 (25.651–87.941)	47.954 (18.064–78.744)	0.732
Fish and shellfish (mg/kcal)^b^	31.295 (23.526–46.635)	28.071 (20.069–35.940)	0.732
Meat (mg/kcal)^b^	44.760 (32.512–58.878)	44.334 (28.299–56.509)	0.455
Eggs (mg/kcal)^b^	14.44 (10.18–27.34)	18.53 (12.93–33.94)	0.174
Dairy products (mg/kcal)^b^	83.29 (27.38–102.69)	71.51 (24.61–102.75)	0.88
Oils and fats (mg/kcal)^a^	6.269 ± 2.745	6.229 ± 2.927	0.887
Confection (mg/kcal)^b^	23.015 (11.670–36.651)	17.095 (9.422–30.137)	0.0369*
Beverages (mg/kcal)^b^	511.91 (282.16–600.25)	386.21 (272.61–515.12)	0.0954
Seasonings and spices (mg/kcal)^b^	105.83 (81.55–145.34)	120.76 (91.62–154.25)	0.144
Sugar and sweeteners (mg/kcal)^b^	2.054 (1.219–3.576)	1.777 (0.970–2.562)	0.0999

Abbreviations: BMI, body mass index; MUFA, monounsaturated fatty acid; NA, not available; PUFA, polyunsaturated fatty acid; SFA, saturated fatty acid; VASI, Vitiligo Area Scoring Index.

^a^ Data provided as mean ± standard deviation, analyzed by paired *t* test.

^b^ Data provided as median (interquartile range), analyzed by Wilcoxon signed rank test.

^c^ Data provided as number (percentage).

^d^ Vitamin A (μg retinoic acid equivalent [RAE]/kcal) is equal to retinol (μg/kcal) + 1/12 × β‐carotene (μg/kcal) + 1/24 × α‐carotene (μg/kcal) + 1/24 × β‐cryptoxanthin (μg/kcal) + 1/24 × other carotenoids (μg/kcal).

^e^ Fisher exact test was used to test the significance of the difference in frequency distribution.

* Significant difference at *p* < 0.05.

** Significant difference at *p* < 0.01.

Sixty age‐ and sex‐matched healthy participants (31 males and 29 females) were chosen as controls from healthy participants composed of recruited volunteers, hospital employees, students, and their family.

### 2.2 Dietary assessment

The dietary habits of the patients and controls were assessed using BDHQ, which is a questionnaire about the patient's diet during the recent 1 month asking the dietary intake of 58 food items.^11^ Estimates of the intakes of foods, energy, and nutrients were calculated using an ad hoc computer algorithm for the BDHQ, based on the Standard Tables of Food Composition in Japan.

### 2.3 Statistical analysis

All statistical analyses were performed using EZR (Saitama Medical Center, Jichi Medical University, Saitama, Japan).^13^ The Shapiro–Wilk test was used to assess the normality of the data distribution. Results are expressed as the mean ± standard deviation for variables with normal distribution or as median (interquartile range) for variables with nonparametric distribution. Differences between patients with vitiligo and controls were analyzed by paired *t* test for variables with normal distribution or by Wilcoxon signed rank test for variables with nonparametric distribution. Differences between the subgroups with versus without vitiligo on each anatomical site, or mild versus moderate to severe vitiligo subgroups were analyzed by Student *t* test for variables with normal distributions or by Mann–Whitney *U*‐test for variables with nonparametric distributions. Fisher's exact test was used to assess the significance of differences in the frequency distributions. Correlations of variables were assessed by Spearman correlation coefficients. Statistical significance was set at *p* < 0.05.

The association of each variable with vitiligo disease, presence of vitiligo on each anatomical site, or moderate to severe vitiligo was evaluated using multivariate logistic regression analyses. The association of each variable with high VASI score was evaluated by linear multivariate regression analyses. These analyses included only the variables with a *p* value < 0.05 in univariate analyses. The analyses for high VASI, vitiligo on each anatomical site, or moderate to severe vitiligo were adjusted for age, sex, and body mass index (BMI). To avoid multicollinearity, variables with a variance inflation factor > 10 were excluded.

## 3 RESULTS

### 3.1 Comparison of dietary habits between patients with vitiligo and controls

The BMI was higher in patients with vitiligo than in controls (Table 1). Patients with vitiligo showed lower intakes of vitamin D, manganese, pulses, and confection, compared with controls (Table 1). According to the multivariate logistic regression analysis (Table 2), vitiligo was associated with high BMI; however, the association with intake of nutrients/foods was not identified.

**TABLE 2:** Association of vitiligo with each variable tested by multivariate logistic regression analysis.

	Odds ratio	95% CI	*p* Value
(Intercept)	0.377	0.0255–5.57	0.478
BMI	1.130	1.02–1.25	0.0208*
Manganese	0.497	0.237–1.04	0.0641
Vitamin D	1.02	0.950–1.11	0.525
Pulses	0.847	0.685–1.05	0.126
Confection	0.983	0.962–1.00	0.117

Abbreviations: BMI, body mass index; CI, confidence interval.

* Statistically significant at *p* < 0.05.

### 3.2 Relationship between VASI and dietary habits in patients with vitiligo

The VASI in males (median, 12 [interquartile range (IQR), 3.86–17]) was higher than that in females (median, 1 [IQR, 0.3–4.2], *p* = 0.000228, by Mann–Whitney *U* test). The VASI inversely correlated with age and intakes of potatoes and vegetables other than green/yellow vegetables (Table 3). The linear multivariate regression analysis showed that high VASI was associated with younger age (Table 4); however, the association with intake of nutrients/foods was not identified.

**TABLE 3:** Correlations of VASI with intakes of nutrients/foods in patients with vitiligo.

	*ρ*	*p* Value
Age (years)	−0.301	0.0196*
Disease duration	0.249	0.0552
BMI	0.174	0.183
Energy intake	0.195	0.136
Nutrients I		
Animal protein	−0.0711	0.589
Plant protein	0.0352	0.79
Animal fat	−0.054	0.682
Plant fat	0.0836	0.525
Carbohydrate	0.0955	0.468
Sodium	0.0171	0.897
Potassium	−0.239	0.0654
Calcium	−0.144	0.272
Magnesium	−0.192	0.142
Phosphorus	−0.166	0.205
Iron	−0.134	0.306
Zinc	−0.133	0.31
Copper	−0.0963	0.464
Manganese	0.0751	0.568
Retinol	0.0626	0.635
β‐Carotene	−0.18	0.169
Vitamin A^a^	−0.0127	0.923
Vitamin D	−0.0568	0.667
α‐Tocopherol	−0.106	0.422
Vitamin K	−0.15	0.253
Vitamin B1	−0.162	0.215
Vitamin B2	−0.118	0.37
Niacin	−0.0665	0.614
Nutrients II		
Vitamin B6	−0.212	0.104
Vitamin B12	0.0764	0.562
Folic acid	−0.116	0.379
Vitamin C	−0.0822	0.532
SFA	0.0421	0.749
MUFA	−0.0191	0.885
*n*‐3PUFA	0.0169	0.898
*n*‐6PUFA	0.0158	0.905
Cholesterol	−0.163	0.214
Alcohol	0.105	0.424
Foods		
Cereals	0.149	0.257
Potatoes	−0.305	0.0179*
Pulses	−0.0977	0.458
Green and yellow vegetables	−0.0357	0.787
Other vegetables	−0.292	0.0235*
Fruit	0.0357	0.786
Fish and shellfish	0.00505	0.969
Meat	0.0487	0.712
Eggs	−0.0728	0.5803
Dairy products	−0.0282	0.831
Oils and fats	0.115	0.3798
Confection	−0.0967	0.462
Beverages	0.131	0.32
Seasonings and spices	0.189502	0.147
Sugar and sweeteners	−0.0977	0.458

*Note*: Correlations between variables were performed using Spearman correlation coefficients.

Abbreviations: BMI, body mass index; MUFA, monounsaturated fatty acid; PUFA, polyunsaturated fatty acid; SFA, saturated fatty acid; VASI, Vitiligo Area Scoring Index.

^a^ Vitamin A (μg retinoic acid equivalent/kcal) is equal to retinol (μg/kcal) + 1/12 × β‐carotene (μg/kcal) + 1/24 × α‐carotene (μg/kcal) + 1/24 × β‐cryptoxanthin (μg/kcal) + 1/24 × other carotenoids (μg/kcal).

* Statistically significant at *p* < 0.05.

**TABLE 4:** Linear multivariate regression analysis for predicting high VASI.

	*β* coefficient	Standard error	*t* Value	*p* Value
(Intercept)	18.4985757	12.11974299	1.5263175	0.13276714
Age	−0.25601115	0.11983597	−2.1363465	0.03720015*
Sex (M = 0, F = 1)	−3.61365303	3.74848933	−0.9640292	0.33932875
BMI	0.48863874	0.42827527	1.1409455	0.25892739
Other vegetables	−0.01975161	0.02969133	−0.6652315	0.50873384
Potatoes	−0.18128582	0.13316598	−1.3613523	0.17905604

Abbreviations: BMI, body mass index; VASI, Vitiligo Area Scoring Index.

* Statistically significant at *p* < 0.05.

In comparison between patients with mild (VASI < 4.25) and moderate to severe (VASI ≥ 4.25) vitiligo, the proportion of males, disease duration, and energy intake were higher in patients with moderate to severe vitiligo compared with those with mild vitiligo (Table S1). The multivariate logistic regression analysis showed that moderate to severe vitiligo was associated with male sex and longer disease duration (Table S2).

### 3.3 Relationship between vitiligo on the head or neck and dietary habits

Patients with vitiligo on the head or neck showed higher intakes of animal protein, animal fat, calcium, vitamin B2, saturated fatty acid (SFA), cholesterol, eggs, and dairy products and lower intake of alcohol, compared with patients without vitiligo (Table 5). The multivariate logistic regression analysis showed that vitiligo on the head or neck was associated with high intake of eggs and dairy products (Table 6).

**TABLE 5:** Intake of nutrients and foods in patients without and with vitiligo on the head or neck.

	Patients without vitiligo (*n* = 12)	Patients with vitiligo (*n* = 48)	*p* Value
Sex			
Male	6	25	1^d^
Female	6	23	
Age (years)^a^	52.67 ± 12.56	54.08 ± 16.13	0.778
Disease duration (years)^b^	5.5 (2.5–10.3)	8.5 (3.0–15.0)	0.304
VASI^b^	2.710 (0.575–8.25)	4.615 (0.788–13.5)	0.705
BMI (kg/m^2^)^b^	23.35 (21.33–25.90)	22.90 (21.38–25.68)	0.882
Energy intake (kcal/day)^b^	1973 (1522–2194)	1680 (1319–2074)	0.292
Nutrients			
Animal protein (% energy)^b^	6.895 (5.468–7.533)	8.035 (7.283–10.735)	0.046*
Plant protein (% energy)^a^	6.2908 ± 0.9278	6.0456 ± 1.4221	0.574
Animal fat (% energy)^a^	11.0875 ± 4.5211	14.1852 ± 4.3796	0.0335*
Plant fat (% energy)^a^	14.0125 ± 3.6136	13.4883 ± 4.2511	0.696
Carbohydrate (% energy)^a^	48.2008 ± 9.2514	49.6815 ± 8.3465	0.593
Sodium (mg/kcal)^b^	2.325 (2.139–2.595)	2.270 (1.928–2.698)	0.919
Potassium (mg/kcal)^b^	1.10 (0.9–1.38)	1.24 (1.043–1.545)	0.275
Calcium (μg/kcal)^b^	204.13 (158.21–277.32)	262.25 (208.73–374.65)	0.0286*
Magnesium (μg/kcal)^b^	123.53 (112.08–142.99)	127.67 (112.26–154.80)	0.616
Phosphorus (μg/kcal)^b^	483.5 (418.5–520.2)	558.1 (469.8–656.9)	0.0627
Iron (μg/kcal)^a^	3.9617 ± 0.9668	4.2794 ± 1.2664	0.421
Zinc (μg/kcal)^a^	4.0967 ± 0.7020	4.4379 ± 0.9223	0.237
Copper (μg/kcal)^b^	0.5942 ± 0.1066	0.5904 ± 0.1327	0.928
Manganese (μg/kcal)^b^	1.365 (1.308–1.658)	1.405 (1.170–1.865)	1
Retinol (μg/kcal)^b^	0.245 (0.1–0.313)	0.19 (0.15–0.37)	0.35
β‐Carotene (μg/kcal)^b^	1.43 (0.958–2.283)	1.52 (0.933–2.363)	0.846
Vitamin A (μg RAE/kcal)^b^ ^,^ ^c^	0.34 (0.25–0.46)	0.40 (0.26–0.57)	0.405
Vitamin D (ng/kcal)^b^	3.99 (3.108–5.338)	4.85 (3.835–7.258)	0.186
α‐Tocopherol (μg/kcal)^a^	3.595 ± 0.958	3.921 ± 1.176	0.379
Vitamin K (μg/kcal)^b^	0.165 (0.128–0.220)	0.165 (0.090–0.213)	0.853
Vitamin B1 (μg/kcal)^a^	0.3800 ± 0.1094	0.4188 ± 0.1101	0.28
Vitamin B2 (μg/kcal)^a^	0.5833 ± 0.1897	0.7552 ± 0.2072	0.0115*
Niacin (μg/kcal)^b^	8.720 (7.228–9.853)	8.795 (7.950–10.640)	0.725
Vitamin B6 (μg/kcal)^b^	0.620 (0.520–0.713)	0.625 (0.558–0.743)	0.585
Vitamin B12 (ng/kcal)^b^	3.845 (2.728–4.1)	3.885 (2.808–5.325)	0.518
Folic acid (μg/kcal)^b^	0.155 (0.125–0.188)	0.175 (0.128–0.223)	0.487
Vitamin C (μg/kcal)^b^	38.48 (31.34–58.15)	49.19 (34.37–64.01)	0.385
SFA (% energy)^a^	6.3558 ± 2.2925	7.8283 ± 2.2392	0.0471*
MUFA (% energy)^a^	9.1608 ± 2.3298	9.8146 ± 2.4114	0.401
*n*‐3PUFA (% energy)^b^	1.24 (1.085–1.36)	1.2 (1.02–1.403)	0.796
*n*‐6PUFA (% energy)^a^	5.2383 ± 1.2263	5.2423 ± 1.3958	0.993
Cholesterol (μg/kcal)^b^	132.38 (125.22–172.43)	197.24 (164.13–283.57)	0.013*
Alcohol (% energy)^b^	8.03 (4.033–20.410)	0.58 (0–9.688)	0.0197*
Foods			
Cereals (mg/kcal)^b^	195.11 (158.23–276.52)	179.42 (152.13–242.39)	0.553
Potatoes (mg/kcal)^b^	19.36 (10.33–25.77)	13.20 (8.95–26.53)	0.524
Pulses (mg/kcal)^b^	2.045 (0.938–2.406)	1.758 (0.970–2.569)	0.993
Green and yellow vegetables (mg/kcal)^b^	42.892 (25.857–65.166)	56.765 (35.949–74.286)	0.375
Other vegetables (mg/kcal)^b^	71.716 (64.289–90.573)	61.147 (41.187–87.545)	0.143
Fruit (mg/kcal)^b^	17.344 (11.161–55.661)	50.238 (29.034–79.746)	0.0807
Fish and shellfish (mg/kcal)^b^	28.691 (20.381–32.309)	27.197 (20.069–41.236)	0.861
Meat (mg/kcal)^b^	48.025 (28.399–56.169)	43.977 (28.299–56.509)	0.455
Eggs (mg/kcal)^b^	12.778 (6.379–18.274)	22.216 (14.336–36.204)	0.00975**
Dairy products (mg/kcal)^b^	17.66 (6.66–53.79)	85.59 (33.88–126.46)	0.00259**
Oils and fats (mg/kcal)^a^	6.3381 ± 2.9559	6.2016 ± 2.9508	0.887
Confection (mg/kcal)^b^	15.575 (10.020–26.264)	17.096 (9.166–31.461)	0.993
Beverages (mg/kcal)^b^	392.22 (347.65–432.23)	381.26 (268.88–565.09)	0.891
Seasonings and spices (mg/kcal)^b^	123.32 (104.032–204.72)	120.76 (87.99–144.84)	0.355
Sugar and sweeteners (mg/kcal)^b^	2.0448 (0.9383–2.4063)	1.7578 (0.9704–2.5687)	0.993

Abbreviations: BMI, body mass index; MUFA, monounsaturated fatty acid; PUFA, polyunsaturated fatty acid; SFA, saturated fatty acid; VASI, Vitiligo Area Scoring Index.

^a^ Data provided as mean ± standard deviation, analyzed by Student *t* test.

^b^ Data provided as median (interquartile range), analyzed by Mann–Whitney *U* test.

^c^ Vitamin A (μg retinoic acid equivalent [RAE]/kcal) is equal to retinol (μg/kcal) + 1/12 × β‐carotene (μg/kcal) + 1/24 × α‐carotene (μg/kcal) + 1/24 × β‐cryptoxanthin (μg/kcal) + 1/24 × other carotenoids (μg/kcal).

^d^ Fisher exact test was used to test the significance of the differences in frequency distribution.

* Significant differences at *p* < 0.05.

** Significant differences at *p* < 0.01.

**TABLE 6:** Association of vitiligo on the head or neck with each variable tested by multivariate logistic regression analysis.

	Odds ratio	95% CI	*p* Value
(Intercept)	0.766	0.00128–457.00	0.935
Age	0.994	0.93–1.06	0.851
Sex (M = 0, F = 1)	0.0817	0.00664–1.01	0.0505
BMI	0.992	0.83–1.19	0.928
Alcohol	1.01	0.919–1.12	0.784
Vitamin B2	2.08	0.00105–4120	0.85
Saturated fatty acid	0.735	0.372–1.46	0.377
Eggs	1.14	1–1.3	0.0447*
Dairy products	1.05	1.01–1.10	0.0196*

*Note*: To avoid multicollinearity, intakes of animal fat, animal protein, calcium, and cholesterol with a variance inflation factor >10 were excluded.Abbreviation: BMI, body mass index; CI, confidence interval.

* Statistically significant at *p* < 0.05.

### 3.4 Relationship between vitiligo on the trunk and dietary habits

Patients with vitiligo on the trunk had higher VASI and younger age, and higher intake of oils and fats compared with patients without vitiligo (Table 7). The logistic regression analysis showed that vitiligo on the trunk was associated with high VASI and high intake of oils and fats (Table 8).

**TABLE 7:** Intake of nutrients and foods in patients without and with vitiligo on the trunk.

	Patients without vitiligo (*n* = 26)	Patients with vitiligo (*n* = 34)	*p* Value
Sex			
Male	10	21	0.117^d^
Female	16	13	
Age (years)^a^	59.615 ± 16.592	49.353 ± 12.964	0.00929**
Disease duration (years)^b^	8 (2–12.5)	7.5 (3–14.5)	0.585
VASI^b^	0.55 (0.20–1.75)	12.5 (5.05–18.5)	2.69 × 10^−9^ **
BMI (kg/m^2^)^b^	22.35 (20.75–25.23)	23.2 (21.43–25.9)	0.474
Energy intake (kcal/day)^b^	1654.4 (1238.0–2034.9)	1868.8 (1494.1–2120.4)	0.147
Nutrients			
Animal protein (% energy)^b^	7.805 (6.803–9.093)	7.925 (6.465–10.525)	0.853
Plant protein (% energy)^a^	6.3573 ± 1.4526	5.8938 ± 1.2208	0.185
Animal fat (% energy)^a^	13.7896 ± 5.3030	13.3944 ± 3.9431	0.742
Plant fat (% energy)^a^	13.4846 ± 4.6450	13.6762 ± 3.7162	0.86
Carbohydrate (% energy)^a^	49.195 ± 8.817	49.531 ± 8.333	0.881
Sodium (mg/kcal)^b^	2.215 (1.785–2.588)	2.315 (2.075–2.683)	0.403
Potassium (mg/kcal)^b^	1.3 (1.115–1.795)	1.135 (1.003–1.450)	0.109
Calcium (μg/kcal)^b^	319.35 (202.07–463.16)	225.49 (190.26–304.52)	0.0877
Magnesium (μg/kcal)^b^	134.33 (115.68–159.12)	121.49 (107.48–143.80)	0.12
Phosphorus (μg/kcal)^b^	566.23 (480.3–682.440)	509.91 (449.14–569.11)	0.151
Iron (μg/kcal)^a^	4.425 ± 1.356	4.056 ± 1.082	0.246
Zinc (μg/kcal)^a^	4.525 ± 1.060	4.251 ± 0.725	0.239
Copper (μg/kcal)^a^	0.6154 ± 0.1427	0.5726 ± 0.1124	0.199
Manganese (μg/kcal)^b^	1.475 (1.218–2.093)	1.39 (1.23–1.548)	0.375
Retinol (μg/kcal)^b^	0.215 (0.153–0.368)	0.21 (0.115–0.365)	0.676
β‐Carotene (μg/kcal)^b^	1.665 (0.88–2.595)	1.27 (0.983–2.293)	0.483
Vitamin A (μg RAE/kcal)^b^ ^,^ ^c^	0.4 (0.28–0.565)	0.375 (0.26–0.533)	0.586
Vitamin D (ng/kcal)^b^	4.655 (3.595–7.873)	4.55 (3.680–6.48)	0.765
α‐Tocopherol (μg/kcal)^a^	4.015 ± 1.403	3.733 ± 0.882	0.345
Vitamin K (μg/kcal)^b^	0.17 (0.12–0.23)	0.155 (0.09–0.208)	0.306
Vitamin B1 (μg/kcal)^a^	0.4342 ± 0.1357	0.3932 ± 0.0836	0.155
Vitamin B2 (μg/kcal)^a^	0.7712 ± 0.2407	0.6824 ± 0.1851	0.1851135
Niacin (μg/kcal)^b^	8.845 (7.425–10.53)	8.72 (8.045–10.413)	0.946
Vitamin B6 (μg/kcal)^b^	0.65 (0.5725–0.8075)	0.615 (0.535–0.6975)	0.387
Vitamin B12 (ng/kcal)^b^	3.82 (2.595–5.48)	3.91 (2.873–5.04)	0.777
Folic acid (μg/kcal)^b^	0.19 (0.13–0.2375)	0.165 (0.1225–0.1975)	0.232
Vitamin C (μg/kcal)^b^	50.075 (33.765–72.845)	47.115 (33.09–55.775)	0.411
SFA (% energy)^a^	7.8485 ± 2.7290	7.2932 ± 1.9349	0.36
MUFA (% energy)^a^	9.5127 ± 2.8180	9.8147 ± 2.0393	0.632
*n*‐3PUFA (% energy)^b^	1.17 (0.9875–1.335)	1.26 (1.03–1.485)	0.451
*n*‐6PUFA (% energy)^a^	5.1077 ± 1.5059	5.3438 ± 1.2381	0.508
Cholesterol (μg/kcal)^b^	187.15 (140.11–282.94)	189.47 (148.40–277.70)	0.959
Alcohol (% energy)^b^	0.08 (0–9.778)	4.56 (0.073–13.588)	0.199
Foods			
Cereals (mg/kcal)^b^	170.60 (139.360–245.76)	190.48 (162.91–247.05)	0.238
Potatoes (mg/kcal)^b^	17.277 (9.565–27.360)	13.141 (8.729–23.289)	0.336
Pulses (mg/kcal)^b^	1.7255 (0.9906–2.4809)	1.8223 (0.9778–2.5631)	0.97
Green and yellow vegetables (mg/kcal)^b^	56.366 (27.641–72.711)	56.166 (29.206–70.445)	0.761
Other vegetables (mg/kcal)^b^	73.553 (48.368–102.458)	57.236 (42.967–82.737)	0.139
Fruit (mg/kcal)^b^	45.621 (17.129–85.713)	49.248 (21.134–72.667)	0.947
Fish and shellfish (mg/kcal)^b^	27.295 (18.561–42.356)	28.360 (21.213–33.563)	1
Meat (mg/kcal)^b^	38.645 (26.84–53.934)	46.147 (30.523–58.368)	0.169
Eggs (mg/kcal)^b^	16.629 (9.388–25.851)	20.357 (14.445–34.575)	0.324
Dairy products (mg/kcal)^b^	81.356 (34.266–131.700)	55.014 (16.293–97.826)	0.121
Oils and fats (mg/kcal)^a^	5.27594 ± 2.44814	6.95763 ± 3.08482	0.0261*
Confection (mg/kcal)^b^	20.002 (10.502–32.403)	13.234 (8.679–22.934)	0.23
Beverages (mg/kcal)^b^	377.4 (210.7–443.8)	399.0 (270.5–628.4)	0.568
Seasonings and spices (mg/kcal)^b^	104.02 (85.81–133.73)	134.53 (93.54–184.14)	0.139
Sugar/sweeteners (mg/kcal)^b^	1.7255 (0.991–2.481)	1.8223 (0.9778–2.5631)	0.97

Abbreviations: BMI, body mass index; MUFA, monounsaturated fatty acid; PUFA, polyunsaturated fatty acid; SFA, saturated fatty acid; VASI, Vitiligo Area Scoring Index.

^a^ Data provided as mean ± standard deviation, analyzed by Student *t* test.

^b^ Data provided as median (interquartile range), analyzed by Mann–Whitney *U* test.

^c^ Vitamin A (μg retinoic acid equivalent [RAE]/kcal) is equal to retinol (μg/kcal) + 1/12 × β‐carotene (μg/kcal) +1/24 × α‐carotene (μg/kcal) + 1/24 × β‐cryptoxanthin (μg/kcal) +1/24 × other carotenoids (μg/kcal).

^d^ Fisher exact test was used to test the significance of the differences in frequency distribution.

* Significant differences at *p* < 0.05.

** Significant differences at *p* < 0.01.

**TABLE 8:** Association of vitiligo on the trunk with each variable tested by multivariate logistic regression analysis.

	Odds ratio	95% CI	*p* Value
(Intercept)	0.0381	0.0000582–24.9	0.323
Age (years)	0.97	0.911–1.03	0.347
Sex (M = 0, F = 1)	9.06	0.881–93.2	0.0638
BMI	0.951	0.774–1.17	0.635
VASI	1.89	1.25–2.86	0.00276**
Oils and fats	1.47	1.02–2.12	0.0411*

Abbreviations: BMI, body mass index; CI, confidence interval; VASI, Vitiligo Area Scoring Index.

* Statistically significant at *p* < 0.05.

** Statistically significant at *p* < 0.01.

### 3.5 Relationship between vitiligo on the upper limbs and dietary habits

Patients with vitiligo on the upper limbs showed higher VASI, higher proportion of males, lower intake of vitamin K, and higher intakes of cereals, fruits, and seasonings/spices, compared with patients without vitiligo (Table 9). The multivariate logistic regression analysis showed that vitiligo on the upper limbs was associated with high VASI and high intake of cereals (Table 10).

**TABLE 9:** Intake of nutrients and foods in patients without and with vitiligo on the upper limbs.

	Patients without vitiligo (*n* = 26)	Patients with vitiligo (*n* = 34)	*p* Value
Sex			
Male	8	23	0.00859**, ^d^
Female	18	11	
Age (years)^a^	55.846 ± 17.426	52.235 ± 13.703	0.372
Disease duration (years)^b^	5.5 (2–10)	10 (4.25–14.75)	0.106
VASI^b^	0.85 (0.225–2.375)	12.5 (4.575–18.5)	6.94 × 10^−7^ **
BMI (kg/m^2^)^b^	22.35 (21.55–23.9)	24.1 (20.85–26.5)	0.27
Energy intake (kcal/day)^b^	1694.2 (1248.0–2110.5)	1741.5 (1494.1–2087.4)	0.491
Nutrients			
Animal protein (% energy)^b^	8.07 (7.135–11.918)	7.725 (6.12–9.06)	0.193
Plant protein (% energy)^a^	6.038 ± 1.311	6.138 ± 1.371	0.775
Animal fat (% energy)^a^	14.348 ± 4.823	12.967 ± 4.295	0.247
Plant fat (% energy)^a^	13.229 ± 4.143	13.871 ± 4.121	0.553
Carbohydrate (% energy)^a^	47.125 ± 8.391	51.114 ± 8.243	0.0705
Sodium (mg/kcal)^b^	2.155 (1.808–2.683)	2.33 (2.045–2.603)	0.612
Potassium (mg/kcal)^b^	1.26 (1.095–1.79)	1.16 (1.003–1.45)	0.146
Calcium (μg/kcal)^b^	311.3 (205.5–463.2)	234.2 (180.9–306.9)	0.077
Magnesium (μg/kcal)^b^	135.85 (115.68–177.55)	122.52 (109.4–137.55)	0.103
Phosphorus (μg/kcal)^b^	583.62 (480.30–757.96)	509.91 (458.86–563.82)	0.077
Iron (μg/kcal)^a^	4.4285 ± 1.4279	4.0532 ± 1.0088	0.238
Zinc (μg/kcal)^a^	4.5565 ± 1.1079	4.2268 ± 0.6567	0.156
Copper (μg/kcal)^a^	0.60385 ± 0.15208	0.58147 ± 0.10546	0.504
Manganese (μg/kcal)^b^	1.335 (1.1025–1.5525)	1.495 (1.3025–2.0875)	0.0746
Retinol (μg/kcal)^b^	0.25 (0.1525–0.37)	0.175 (0.115–0.35)	0.303
β‐Carotene (μg/kcal)^b^	1.705 (1.005–2.195)	1.045 (0.8975–2.4925)	0.512
Vitamin A (μg RAE/kcal)^b^ ^,^ ^c^	0.44 (0.28–0.575)	0.355 (0.245–0.4825)	0.269
Vitamin D (ng/kcal)^b^	4.985 (3.765–14.6575)	4.38 (3.565–5.63)	0.325
α‐Tocopherol (μg/kcal)^a^	4.012692 ± 1.262203	3.735294 ± 1.031742	0.353
Vitamin K (μg/kcal)^b^	0.205 (0.1225–0.26)	0.145 (0.0925–0.19)	0.0358*
Vitamin B1 (μg/kcal)^a^	0.42692 ± 0.12890	0.39882 ± 0.09361	0.332
Vitamin B2 (μg/kcal)^a^	0.7808 ± 0.2276	0.675 ± 0.1935	0.0568
Niacin (μg/kcal)^b^	9.22 (6.97–13.47)	8.66 (7.973–9.5675)	0.26
Vitamin B6 (μg/kcal)^b^	0.655 (0.5625–0.905)	0.605 (0.535–0.6925)	0.197
Vitamin B12 (ng/kcal)^b^	4.07 (2.615–7.808)	3.72 (3.01–4.84)	0.531
Folic acid (μg/kcal)^b^	0.17 (0.13–0.23)	0.17 (0.1225–0.205)	0.415
Vitamin C (μg/kcal)^b^	44.395 (32.333–69.518)	49.185 (33.753–62.675)	0.728
SFA (% energy)^a^	7.783462 ± 2.428927	7.342941 ± 2.22876	0.468
MUFA (% energy)^a^	9.66769 ± 2.33925	9.69618 ± 2.46363	0.964
*n*‐3PUFA (% energy)^b^	1.24 (0.9975–1.59)	1.125 (1.03–1.3775)	0.328
*n*‐6PUFA (% energy)^a^	5.154231 ± 1.364461	5.308235 ± 1.361958	0.666
Cholesterol (μg/kcal)^b^	217.67 (165.60–317.06)	172.85 (132.19–250.72)	0.0548
Alcohol (% energy)^b^	0.505 (0–13.378)	3.835 (0–10.57)	0.691
Foods			
Cereals (mg/kcal)^b^	167.45 (133.67–208.00)	209.79 (166.34–256.69)	0.00815**
Potatoes (mg/kcal)^b^	18.728 (12.646–26.303)	11.514 (7.332–23.783)	0.0591
Pulses (mg/kcal)^b^	1.8348 (1.2028–2.5597)	1.7601 (0.8163–2.5161)	0.445
Green and yellow vegetables (mg/kcal)^b^	55.347 (30.6529–62.195)	57.610 (27.325–71.548)	0.935
Other vegetables (mg/kcal)^b^	69.104 (46.776–102.458)	63.161 (42.967–84.995)	0.25
Fruit (mg/kcal)^b^	27.470 (12.955–55.504)	53.929 (35.442–86.659)	0.0459*
Fish and shellfish (mg/kcal)^b^	28.402 (18.561–67.607)	26.709 (20.527–32.758)	0.371
Meat (mg/kcal)^b^	40.923 (27.221–54.296)	47.076 (29.239–57.7835)	0.402
Eggs (mg/kcal)^b^	23.532 (14.904–34.575)	15.049 (12.283–29.557)	0.215
Dairy products (mg/kcal)^b^	84.201 (34.266–111.876)	61.786 (15.318–100.718)	0.15
Oils and fats (mg/kcal)^a^	5.8505 ± 3.1124	6.5182 ± 2.7895	0.386
Confection (mg/kcal)^b^	20.105 (12.634–32.404)	13.234 (7.931–25.312)	0.0699
Beverages (mg/kcal)^b^	383.43 (271.39–500.08)	386.21 (278.47–527.44)	0.75
Seasonings and spices (mg/kcal)^b^	101.98 (77.27–137.13)	133.53 (103.60–184.14)	0.0328*
Sugar/sweeteners (mg/kcal)^b^	1.8348 (1.2028–2.5597)	1.76013 (0.8163–2.5161)	0.445

Abbreviations: BMI, body mass index; MUFA, monounsaturated fatty acid; PUFA, polyunsaturated fatty acid; SFA, saturated fatty acid; VASI, Vitiligo Area Scoring Index.

^a^ Data provided as mean ± standard deviation, analyzed by Student *t* test.

^b^ Data provided as median (interquartile range), analyzed by Mann–Whitney *U* test.

^c^ Vitamin A (μg retinoic acid equivalent [RAE]/kcal) is equal to retinol (μg/kcal) + 1/12 × β‐carotene (μg/kcal) + 1/24 × α‐carotene (μg/kcal) + 1/24 × β‐cryptoxanthin (μg/kcal) + 1/24 × other carotenoids (μg/kcal).

^d^ Fisher exact test was used to test the significance of the differences in frequency distribution.

* Significant differences at *p* < 0.05.

** Significant differences at *p* < 0.01.

**TABLE 10:** Association of vitiligo on the upper limbs with each variable tested by multivariate logistic regression analysis.

	Odds ratio	95% CI	*p* Value
(Intercept)	0.00000252	5.33 × 10^−11^–0.119	0.0189
Age (years)	1.03	0.97–1.1	0.311
Sex (M = 0, F = 1)	4.43	0.428–45.9	0.212
BMI	1.22	0.954–1.55	0.114
VASI	1.76	1.17–2.65	0.00668**
Vitamin K	0.0000168	2.61 × 10^−11^–10.9	0.107
Cereals	1.03	1.01–1.04	0.0116*
Seasonings and spices	1.0	0.990–1.02	0.654

*Note*: To avoid multicollinearity, intake of fruits with a variance inflation factor >10 was excluded. Abbreviations: BMI, body mass index; VASI, Vitiligo Area Scoring Index.

* Statistically significant at *p* < 0.05.

** Statistically significant at *p* < 0.01.

### 3.6 Relationship between vitiligo on the lower limbs and dietary habits

Patients with vitiligo on the lower limbs had higher VASI, higher proportion of males, and higher intake of vitamin B12, compared with patients without vitiligo (Table 11). The multivariate logistic regression analysis showed that vitiligo on the lower limbs was associated with male sex (Table 12).

**TABLE 11:** Intake of nutrients and foods in patients without and with vitiligo on the lower limbs.

	Patients without vitiligo (*n* = 29)	Patients with vitiligo (*n* = 31)	*p* Value
Sex			
Male	8	23	6.46 × 10^−4^ **, ^d^
Female	21	8	
Age (years)^a^	55.828 ± 17.109	51.903 ± 13.605	0.328
Disease duration (years)^b^	5 (3.0–10.0)	10 (3.5–15.5)	0.0868
VASI^b^	0.8 (0.3–3.1)	13 (5.3–18.0)	1.68 × 10^−6^ **
BMI (kg/m^2^)^b^	23 (21.7–26.8)	22.8 (20.6–25.3)	0.359
Energy intake (kcal/day)^b^	1547.4 (1249–1963)	1864.8 (1549–2182.2)	0.111
Nutrients			
Animal protein (% energy)^b^	7.89 (6.7–9.93)	7.82 (6.665–10.545)	0.736
Plant protein (% energy)^a^	6.0979 ± 1.3324	6.0916 ± 1.3588	0.986
Animal fat (% energy)^a^	13.7114 ± 4.3644	13.4294 ± 4.7746	0.812
Plant fat (% energy)^a^	13.5114 ± 3.6074	13.6697 ± 4.5859	0.883
Carbohydrate (% energy)^a^	49.9372 ± 8.9653	48.8690 ± 8.1017	0.63
Sodium (mg/kcal)^b^	2.16 (1.95–2.55)	2.34 (2.015–2.675)	0.46
Potassium (mg/kcal)^b^	1.24 (1.05–1.56)	1.19 (1.015–1.47)	0.706
Calcium (μg/kcal)^b^	244.11 (199.31–359.87)	245.59 (205.965–343.67)	0.907
Magnesium (μg/kcal)^b^	127.21 (114.42–152.73)	124.55 (108.015–155.64)	0.93
Phosphorus (μg/kcal)^b^	557.12 (469.98–608.17)	544.33 (455.11–633.87)	0.895
Iron (μg/kcal)^a^	4.2362 ± 1.2835	4.1968 ± 1.1616	0.901
Zinc (μg/kcal)^a^	4.3903 ± 0.8673	4.3503 ± 0.9208	0.863
Copper (μg/kcal)^a^	0.59552 ± 0.14456	0.58710 ± 0.11052	0.8
Manganese (μg/kcal)^b^	1.37 (1.11–1.83)	1.48 (1.29–2.015)	0.27
Retinol (μg/kcal)^b^	0.16 (0.13–0.31)	0.26 (0.155–0.395)	0.122
β‐Carotene (μg/kcal)^b^	1.53 (0.99–2.24)	1.44 (0.915–2.535)	0.767
Vitamin A (μg RAE/kcal)^b^ ^,^ ^c^	0.34 (0.22–0.44)	0.45 (0.285–0.585)	0.127
Vitamin D (ng/kcal)^b^	4.1 (3.14–5.28)	5.51 (4.03–7.055)	0.151
α‐Tocopherol (μg/kcal)^a^	3.9214 ± 1.2968	3.7939 ± 0.9792	0.668
Vitamin K (μg/kcal)^b^	0.16 (0.11–0.23)	0.18 (0.09–0.21)	0.784
Vitamin B1 (μg/kcal)^a^	0.42 ± 0.1165	0.4026 ± 0.1052	0.545
Vitamin B2 (μg/kcal)^a^	0.71724 ± 0.19966	0.72419 ± 0.22939	0.901
Niacin (μg/kcal)^b^	8.42 (7.27–10)	9.25 (8.24–10.77)	0.222
Vitamin B6 (μg/kcal)^b^	0.65 (0.53–0.74)	0.62 (0.555–0.74)	0.97
Vitamin B12 (ng/kcal)^b^	2.81 (2.59–5.02)	4.07 (3.335–5.42)	0.0265*
Folic acid (μg/kcal)^b^	0.16 (0.12–0.21)	0.17 (0.135–0.225)	0.423
Vitamin C (μg/kcal)^b^	38.64 (30.28–63.72)	51.22 (37.12–64.72)	0.233
SFA (% energy)^a^	7.6628 ± 2.4312	7.4132 ± 2.2199	0.679
MUFA (% energy)^a^	9.6459 ± 2.2573	9.7194 ± 2.5453	0.906
*n*‐3PUFA (% energy)^b^	1.14 (1.02–1.39)	1.25 (1.035–1.405)	0.478
*n*‐6PUFA (% energy)^a^	5.1907 ± 1.2234	5.2890 ± 1.4839	0.781
Cholesterol (μg/kcal)^b^	178.25 (150.18–307.65)	190.73 (131.38–276.83)	0.527
Alcohol (% energy)^b^	0.29 (0–10.52)	5.03 (0–12.845)	0.268
Foods			
Cereals (mg/kcal)^b^	180.0 (1140.26–252.54)	186.45 (156.97–239.78)	0.736
Potatoes (mg/kcal)^b^	16.829 (11.048–25.908)	12.928 (8.253–26.268)	0.321
Pulses (mg/kcal)^b^	1.8952 (1.1828–2.6482)	1.6669 (0.8499–2.3859)	0.394
Green and yellow vegetables (mg/kcal)^b^	55.0398 (27.8360–73.0619)	59.246 (39.288–71.092)	0.703
Other vegetables (mg/kcal)^b^	69.561 (47.441–93.314)	62.609 (43.089–84.901)	0.471
Fruit (mg/kcal)^b^	37.526 (11.888–59.721)	52.707 (32.734–85.172)	0.2
Fish and shellfish (mg/kcal)^b^	22.317 (17.501–34.361)	30.964 (24.013–38.280)	0.0554
Meat (mg/kcal)^b^	44.502 (26.961–54.335)	44.166 (28.639–58.33)	0.394
Eggs (mg/kcal)^b^	21.795 (13.886–33.622)	15.236 (12.439–32.831)	0.41
Dairy products (mg/kcal)^b^	77.461 (26.08–102.626)	59.663 (21.945–101.359)	0.646
Oils and fats (mg/kcal)^a^	6.0348 ± 2.8251	6.4105 ± 3.0547	0.623
Confection (mg/kcal)^b^	18.756 (235.167–507.471)	15.8 (8.018–25.432)	0.336
Beverages (mg/kcal)^b^	394.766 (235.167–507.471)	379.78 (285–570.797)	0.577
Seasonings and spices (mg/kcal)^b^	113.25 (86.98–138.38)	132.50 (94.57–170.28)	0.471
Sugar/sweeteners (mg/kcal)^b^	1.8952 (1.1828–2.6482)	1.6669 (0.8499–2.3859)	0.394

Abbreviations: BMI, body mass index; MUFA, monounsaturated fatty acid; PUFA, polyunsaturated fatty acid; RAE, retinoic acid equivalent; SFA, saturated fatty acid; VASI, Vitiligo Area Scoring Index.

^a^ Data provided as mean ± standard deviation, analyzed by Student *t* test.

^b^ Data provided as median (interquartile range), analyzed by Mann–Whitney *U* test.

^c^ Vitamin A (μg retinoic acid equivalent [RAE]/kcal) is equal to retinol (μg/kcal) + 1/12 × β‐carotene (μg/kcal) + 1/24 × α‐carotene (μg/kcal) + 1/24 × β‐cryptoxanthin (μg/kcal) + 1/24 × other carotenoids (μg/kcal).

^d^ Fisher exact test was used to test the significance of the differences in frequency distribution.

* Significant differences at *p* < 0.05.

** Significant differences at *p* < 0.01.

**TABLE 12:** Association of vitiligo on the lower limbs with each variable tested by multivariate logistic regression analysis.

	Odds ratio	95% CI	*p* Value
(Intercept)	18.9	0.248–1440	0.184
Age (years)	0.999	0.955–1.04	0.952
Sex (M = 0, F = 1)	0.123	0.0311–0.483	0.0027*
BMI	0.885	0.756–1.04	0.127
VASI	1.05	0.986–1.12	0.128
Vitamin B12	1.14	0.932–1.4	0.2

Abbreviations: BMI, body mass index; CI, confidence interval; VASI, Vitiligo Area Scoring Index.

* Statistically significant at *p* < 0.01.

### 3.7 Relationship between vitiligo on the hands or feet and dietary habits

Patients with vitiligo on the hands or feet had higher VASI, longer disease duration, higher proportion of males, higher BMI, and higher intakes of energy and meat, compared with patients without vitiligo (Table 13). The multivariate logistic regression analysis showed that vitiligo on the hands or feet was associated with high VASI and high BMI (Table 14).

**TABLE 13:** Intake of nutrients and foods in patients without and with vitiligo on the hands or feet.

	Patients without vitiligo (*n* = 28)	Patients with vitiligo (*n* = 32)	*p* Value
Sex			
Male	7	24	2.23 × 10^−4^ **, ^d^
Female	21	8	
Age (years)^a^	57.786 ± 15.81	50.313 ± 14.365	0.06
Disease duration (years)^b^	5 (1.75–10)	10 (5–17.75)	0.00687**
VASI^b^	0.775 (0.275–2.355)	13 (5.1325–19)	2.57 × 10^−8^ **
BMI (kg/m^2^)^b^	22.35 (18.425–24.15)	24.4 (21.55–26.95)	0.0311*
Energy intake (kcal/day)^b^	1525.8 (1244.4–1990.6)	1866 (1602.8–2125.8)	0.041*
Nutrients			
Animal protein (% energy)^b^	7.375 (6.3725–11.04)	8.035 (7.235–9.955)	0.591
Plant protein (% energy)^a^	6.090714 ± 1.304667	6.098125 ± 1.381195	0.983
Animal fat (% energy)^a^	13.11071 ± 4.78796	13.96375 ± 4.35748	0.473
Plant fat (% energy)^a^	12.77464 ± 4.31046	14.30937 ± 3.84721	0.15
Carbohydrate (% energy)^a^	49.49286 ± 9.06722	49.29125 ± 8.06499	0.928
Sodium (mg/kcal)^b^	2.22 (1.835–2.6875)	2.325 (2.075–2.6125)	0.625
Potassium (mg/kcal)^b^	1.235 (1.035–1.61)	1.205 (1.0175–1.46)	0.7
Calcium (μg/kcal)^b^	273.035 (195.03–428.81)	238.22 (205.55–312.33)	0.475
Magnesium (μg/kcal)^b^	128.95 (112.14–157.81)	124.11 (112.08–142.39)	0.591
Phosphorus (μg/kcal)^b^	545.39 (425.85–733.2)	546.69 (469.79–565.61)	0.601
Iron (μg/kcal)^a^	4.23107 ± 1.38701	4.2025 ± 1.05761	0.928
Zinc (μg/kcal)^a^	4.39321 ± 1.0242	4.34906 ± 0.76559	0.85
Copper (μg/kcal)^a^	0.601786 ± 0.150949	0.581875 ± 0.103408	0.549
Manganese (μg/kcal)^b^	1.35 (1.095–1.8325)	1.455 (1.295–1.9675)	0.296
Retinol (μg/kcal)^b^	0.19 (0.11–0.3825)	0.25 (0.1475–0.35)	0.953
β‐Carotene (μg/kcal)^b^	1.635 (0.8125–2.2575)	1.475 (0.995–2.5275)	0.614
Vitamin A (μg RAE/kcal)^b^ ^,^ ^c^	0.37 (0.2125–0.59)	0.4 (0.3075–0.4675)	0.906
Vitamin D (ng/kcal)^b^	4.59 (2.9525–13.7575)	4.55 (4.035–6.0525)	0.818
α‐Tocopherol (μg/kcal)^a^	3.792857 ± 1.333125	3.910312 ± 0.948682	0.693
Vitamin K (μg/kcal)^b^	0.175 (0.1175–0.23)	0.16 (0.0975–0.2025)	0.358
Vitamin B1 (μg/kcal)^a^	0.403929 ± 0.124435	0.417188 ± 0.097625	0.646
Vitamin B2 (μg/kcal)^a^	0.736429 ± 0.225392	0.707188 ± 0.20565	0.601
Niacin (μg/kcal)^b^	8.75 (6.8–11.7)	8.765 (8.1925–9.8875)	0.906
Vitamin B6 (μg/kcal)^b^	0.6 (0.5275–0.84)	0.64 (0.575–0.71)	0.876
Vitamin B12 (ng/kcal)^b^	3.37 (2.5875–5.8925)	3.995 (3.265–5.02)	0.394
Folic acid (μg/kcal)^b^	0.17 (0.1175–0.23)	0.17 (0.13–0.2025)	0.882
Vitamin C (μg/kcal)^b^	44.555 (31.4575–64.4625)	49.03 (35.1825–63.06)	0.512
SFA (% energy)^b^	7.243214 ± 2.579858	7.788125 ± 2.048898	0.366
MUFA (% energy)^a^	9.066786 ± 2.36123	10.22375 ± 2.318104	0.0608
*n*‐3PUFA (% energy)^b^	1.2 (0.90–1.515)	1.21 (1.0375–1.3925)	0.711
*n*‐6PUFA (% energy)^a^	4.922143 ± 1.384877	5.520937 ± 1.282244	0.0874
Cholesterol (μg/kcal)^b^	193.39 (147.13–294.64)	186.78 (144.04–277.15)	0.797
Alcohol (% energy)^b^	0.635 (0–14.94)	3.835 (0–8.99)	0.79
Foods			
Cereals (mg/kcal)^b^	174.33 (142.91–258.68)	187.54 (152.53–242.39)	0.797
Potatoes (mg/kcal)^b^	17.277 (10.992–26.711)	12.72 (8.417–25.77)	0.289
Pulses (mg/kcal)^b^	1.7859 (1.0578–2.5391)	1.7767 (0.8836–2.5619)	0.73
Green and yellow vegetables (mg/kcal)^b^	43.706 (25.861–60.289)	58.372 (43.0527–79.859)	0.112
Other vegetables (mg/kcal)^b^	71.716 (51.84–101.81)	55.48 (33.74–89.77)	0.122
Fruit (mg/kcal)^b^	39.303 (15.089–58.45)	50.238 (33.741–89.766)	0.15
Fish and shellfish (mg/kcal)^b^	24.958 (17.632–55.524)	29.343 (21.384–33.401)	0.594
Meat (mg/kcal)^b^	40.923 (26.718–47.65)	53.255 (30.456–58.412)	0.0315*
Eggs (mg/kcal)^b^	19.34 (12.545–29.892)	17.207 (12.953–35.55)	0.947
Dairy products (mg/kcal)^b^	80.794 (31.5497–101.516)	61.786 (17.174–108.893)	0.529
Oils and fats (mg/kcal)^a^	5.5528 ± 3.1265	6.8205 ± 2.6495	0.0944
Confection (mg/kcal)^b^	18.049 (10.476–24.228)	13.677 (8.105–33.082)	0.62
Beverages (mg/kcal)^b^	383.43 (251.07–547.4)	386.21 (294.69–488.25)	0.82
Seasonings and spices (mg/kcal)^b^	110.765 (87.740–227.505)	129.78 (95.602–141.71)	0.901
Sugar/sweeteners (mg/kcal)^b^	1.7859 (1.0578–2.5391)	1.7767 (0.8836–2.5619)	0.73

Abbreviations: BMI, body mass index; MUFA, monounsaturated fatty acid; PUFA, polyunsaturated fatty acid; SFA, saturated fatty acid; VASI, Vitiligo Area Scoring Index.

^a^ Data provided as mean ± standard deviation, analyzed by Student *t* test.

^b^ Data provided as median (interquartile range), analyzed by Mann–Whitney *U* test.

^c^ Vitamin A (μg retinoic acid equivalent [RAE]/kcal) is equal to retinol (μg/kcal) + 1/12 × β‐carotene (μg/kcal) + 1/24 × α‐carotene (μg/kcal) + 1/24 × β‐cryptoxanthin (μg/kcal) + 1/24 × other carotenoids (μg/kcal).

^d^ Fisher exact test was used to test the significance of the differences in frequency distribution.

* Significant differences at *p* < 0.05.

** Significant differences at *p* < 0.01.

**TABLE 14:** Association of vitiligo on the hands or feet with each variable tested by multivariate logistic regression analysis.

	Odds ratio	95% CI	*p* Value
(Intercept)	0.00000637	4.54 × 10^−10^–0.0893	0.0141
Age (years)	0.969	0.891–1.05	0.466
Sex (M = 0, F = 1)	0.297	0.0317–2.77	0.287
BMI	1.5	1.11–2.03	0.0088*
VASI	1.56	1.11–2.19	0.00986*
Disease duration	1.21	0.986–1.48	0.0677
Energy intake	1	0.975–1.04	0.589
Meat	1.01	0.975–1.04	0.6

Abbreviations: BMI, body mass index; CI, confidence interval; VASI, Vitiligo Area Scoring Index.

* Statistically significant at *p* < 0.01.

## 4 DISCUSSION

Patients with vitiligo had higher BMI compared with controls, and high BMI was a predictive factor for vitiligo. High BMI in patients was also a predictive factor for vitiligo on the hands or feet. These results indicate a close relationship between obesity and vitiligo, and are consistent with the previous studies showing that patients with vitiligo are associated with obesity or metabolic syndromes more frequently than healthy controls.^14^, ^15^, ^16^ These findings indicate that control of obesity and/or metabolic syndromes may prevent the development or exacerbation of vitiligo. Obesity is associated with hyperplasia of visceral adipose tissues, causing hypoxia of adipocytes.^17^ The hypoxia activates NADPH oxidase and triggers ROS generation in visceral adipose tissues,^18^ extending to systemic oxidative stress, triggering the development of vitiligo. The hypoxia of adipocytes promotes their release of chemokines CCL5, CCL2, and CXCL10, which induce the recruitment and accumulation of TNF‐α– or IL‐1–producing proinflammatory M1 macrophages, IFN‐γ–producing CD4+ or CD8+ T cells, and Th17 cells, making the adipose tissue a reservoir of inflammatory immune cells.^17^ The hypoxia and ROS induce adipocytes to secrete proinflammatory adipokines such as TNF‐α or leptin, which may circulate and reach the skin and may induce autoimmune responses to melanocytes. Especially, leptin might promote the progression of vitiligo^19^ by enhancing the cytotoxic function of CD8+ T cells. In mice, *LEPTIN* deficiency ameliorated the development of vitiligo and reduced the expression of *Cxcl9*, *Gzmb*, *Ifng*, and *Mx1* in vitiligo lesions.^18^

Patients with vitiligo showed significantly lower intakes of several nutrients/foods, compared with controls, although the significance was lost in multivariate analysis. First, the intake of manganese was reduced in patients with vitiligo. Manganese is rich in grains, rice, soybeans, nuts, vegetables, fruits, and tea, and is a constituent of antioxidant enzyme manganese superoxide dismutase. Lack of manganese may reduce manganese superoxide dismutase activity, leading to the accumulation of ROS in melanocytes, promoting cell death.^20^, ^21^, ^22^ Further, the reduced intake of manganese may be related to the higher BMI in patients with vitiligo since dietary manganese may prevent obesity.^22^, ^23^ Supplementation of manganese reduces abdominal fat accumulation by decreasing lipoprotein lipase and malate dehydrogenase activities.^24^

Second, the intake of pulses was reduced in patients with vitiligo. Pulses, especially soybean, are enriched with bioactive peptides, isoflavones, or saponins, which inhibit the production of ROS and proinflammatory cytokines, such as TNF‐α and IL‐1β, by inhibiting nuclear factor‐κB.^25^ The deficiency of dietary pulses may promote the oxidative stress and inflammation, triggering vitiligo.

Third, the intake of vitamin D was reduced in patients with vitiligo. The results may be related to previous studies showing the reduced serum level of 25‐hydroxyvitamin D in patients with vitiligo compared with controls.^8^, ^9^ Vitamin D analog tacalcitol scavenges ROS and suppresses the ROS‐induced apoptosis of melanocytes.^26^ The active form of vitamin D, 1,25‐dihydroxyvitamin D3, induces melanogenesis in melanocytes.^27^ The deficiency in dietary vitamin D may lose its protective effects for vitiligo.

Fourth, the intake of confection is reduced in patients with vitiligo. The result is opposite to our expectation since the reduced intake of sugar‐rich confection might prevent hyperglycemia, and rather suppress the onset of vitiligo. The reduction of glucose levels suppresses the production of IFN‐γ in CD8+ T cells.^28^ The reduced intake of confection in adult Japanese patients with vitiligo may reflect their food preference, or their intention to evade sugar‐rich confection to reduce the risk of obesity. Alternatively, reduced intake of confection might generate hypoglycemia, which stimulates the generation of mitochondrial ROS^29^ or cellular heat shock–related responses and increases blood IL‐6 levels,^30^ triggering vitiligo.

The VASI negatively correlated with the intakes of potatoes and vegetables other than green/yellow vegetables, although the significance disappeared in multivariate analysis. These foods abundantly contain dietary fibers,^31^ which are fermented into short‐chain fatty acids, such as butyrate or propionate in the gut.^32^ Short‐chain fatty acids promote the differentiation of naïve CD4+ T cells into Foxp3+ Tregs,^32^ which suppress the proliferation of autoreactive CD8+ or CD4+ effector T cells targeting melanocytes. Previous studies also reported that impairment of Treg activity in patients with vitiligo might play a disease‐progressive role.^33^ The reduced intake of potatoes and vegetables other than green/yellow vegetables may lead to the deficiency of short‐chain fatty acids, reducing Treg activity, resultantly accelerating autoimmune responses in vitiligo lesions.

The patients with moderate to severe vitiligo showed significantly higher energy intake compared with patients with mild vitiligo, although the significance disappeared in multivariate analysis. A high‐calorie diet promotes apoptosis of murine aortic endothelial cells via induction of ROS and endoplasmic reticulum stress,^34^ and might similarly induce apoptosis of melanocytes in vitiligo lesions.

The presence of vitiligo on different anatomical sites was associated with the increased or decreased intakes of several nutrients/foods. The altered intake may be related to the development of vitiligo on each site. However, the causal relationship between each nutrient/food and localization of vitiligo is unknown, and should be further elucidated. Vitiligo on the head or neck was associated with high intake of eggs and dairy products. The patients with vitiligo on the head or neck also showed significantly higher intakes of cholesterol and SFA compared with those without vitiligo on this site, although the significance was lost in multivariate analysis. Since cholesterol or SFA is enriched in eggs or dairy products, respectively, increased intake of those foods may accelerate proinflammatory effects of cholesterol and SFA. Excess intake of cholesterol induces the generation of ROS by activating NADPH oxidase while suppressing antioxidant enzymes, SOD, or glutathione peroxidase.^35^ Further, hypercholesterolemia induced by excess dietary cholesterol is associated with the increased serum level of IL‐15,^36^, ^37^ which potentiates the survival of T_RM_ cells attacking melanocytes. Excess dietary cholesterol increased IL‐15 messenger RNA levels in the skin of young grass carp infected with *Aeromonas hydrophila*.^38^ These effects of excess dietary cholesterol may trigger vitiligo on the head or neck.

SFAs such as palmitic acid suppress autophagy in hepatocytes, decreasing the levels of Nrf2, inducing ferroptosis of hepatocytes.^39^, ^40^ In a similar manner, excess intake of dietary SFA may promote ferroptosis of melanocytes in patients with vitiligo. Further, SFAs promote the differentiation of naïve CD4+ T cells toward Th1 or Th17 cells,^41^ while suppressing the differentiation into Tregs.^42^ These effects of excess dietary SFA may favor the development of vitiligo on the head or neck.

Vitiligo on the trunk was associated with high intake of oils and fats. The excess intake of oils and fats such as margarine, cooking oil, or lard may increase serum levels of cholesterol and/or low‐density lipoprotein cholesterol, which may induce ROS generation^43^ and production of proinflammatory cytokines such as TNF‐α or IFN‐γ in visceral adipose tissues and production of IL‐15 in the skin.^44^ These effects of excess dietary oils/fats might trigger vitiligo on the trunk.

Vitiligo on the upper limbs was associated with high intake of cereals. Among cereals, refined grains such as white bread or white rice are low‐quality carbohydrates with a high glycemic index. The excess intake of refined grains rapidly increases plasma glucose levels, leading to hyperglycemia,^31^ which induces ROS generation by activating protein kinase C and hexosamine and sorbitol pathways.^29^ Further, excess glucose interacts with proteins or lipids and produces advanced glycation end products^29^ that enhance IFN‐γ production of CD8+ T cells.^45^, ^46^ These effects of excess dietary cereals might trigger vitiligo on the upper limbs.

High VASI was associated with younger age. The VASI was higher in males than in females. Further, male sex was a predictive factor for moderate to severe vitiligo and vitiligo on the lower limbs. Moderate to severe vitiligo was also associated with longer disease duration, indicating the progressiveness of vitiligo possibly via T_RM_ cells. The present results indicate that the severity of vitiligo may be associated with younger age and male sex. The association with male sex might reflect the influence of sex hormones; estrogen stimulates melanogenesis by increasing the expression of tyrosinase,^47^ while testosterone suppresses tyrosinase activity,^48^ indicating a protective or progressive role of estrogen or testosterone for vitiligo, respectively. Regarding the association with younger age, Giri et al. reported that Tregs from patients with vitiligo of early onset (≤ 20 years) showed lower activities to suppress proliferation of CD8+ or CD4+ T cells and reduced expression of transforming growth factor β and nuclear factor of activated T cells 1, compared with patients with later‐onset vitiligo (> 40 years).^33^ The results indicate that reduced Treg activities in younger patients may be related to the higher severity of vitiligo. Mu et al. also reported that childhood vitiligo of early onset (< 3 years) was associated with more extensive and progressive course compared with that of later onset (3–18 years).^49^ Mahajan et al. reported that patients with vitiligo with early onset (< 10 years) are associated with a higher rate of vitiligo with > 10% body surface area involvement and higher proportion of males, compared with patients with later onset (≥ 10 years).^50^ Those studies indicate the association of severity with early childhood onset and of male preponderance with early onset. The patients in the present study mostly showed adult‐onset vitiligo (≥ 20 years); however, the proportion of males in the youngest (< 48 years), middle (48–59 years), or oldest (≥ 60 years) age groups was 65%, 45%, or 45%, respectively, indicating a male preponderance in the youngest age group, although not statistically significant (*p* = 0.3809, by Fisher's exact test). The relationship of age and sex with severity of vitiligo should further be examined by extensive studies using a larger and multiracial cohort with a wide range of age.

This study has several limitations; first, the sample size was small. Second, the cohort consisted of Japanese patients only. Third, patients with segmental vitiligo were not studied. Last, BDHQ might overlook the intake of micronutrients from supplements.

In conclusion, vitiligo was associated with high BMI. The high VASI was associated with younger age. Moderate to severe vitiligo was associated with male sex and longer disease duration. The vitiligo on each anatomical site showed the following association: high intake of eggs and dairy products and high VASI on the head/neck; high intake of oils/fats and high VASI on the trunk; high intake of cereals and high VASI on the upper limbs; male sex and high VASI on the lower limbs; and high BMI and high VASI on the hands/feet. The control of obesity might have prophylactic or therapeutic effects on vitiligo.

## CONFLICT OF INTEREST STATEMENT

H. S. is an editorial board member of the *Journal of Dermatology* and a coauthor of the current article. To minimize bias, he was excluded from all editorial decision‐making related to the acceptance of this article for publication. There are no other conflicts of interest to be declared.

## Supporting information


Data S1.
