# Supplementary material for: Dietary habits in adult Japanese patients with vitiligo
Source: J Dermatol. 2024 Feb 29;51(4):491–508. doi: 10.1111/1346-8138.17163 (PMC11484454; doi:10.1111/1346-8138.17163)
Supplement: Supplementary file 1 — Data S1. [file JDE-51--s001.docx]

| Supplemental table 1. The intake of nutrients and foods in patients with mild or moderate-to-severe vitiligo | | | | | |  |  |
| --- | --- | --- | --- | --- | --- | --- | --- |
|  | | Mild (*n* = 30) | Moderate-to-severe  (*n* = 30) | | *ｐ*values | |  |
| Vitiligo area severity index^a^ | | 0.6125 (0.225-1) | | 14 (6.725-19) | 2.94 x 10^-11**^ | | |
| Sex | Male | 8 | | 23 | 0.00799^**d^ | | |
|  | Female | 22 | | 7 |  | | |
| Age (years) ^b^ | | 56.33 ± 15.00 | | 51.27 ± 15.61 | 0.205 | | |
| Disease duration (years)^a^ | | 5.5 (1.25-10) | | 10 (4.25-19.25) | 0.00799^**^ | | |
| Body mass index (kg/m^2^) ^a^ | | 22.55 (21.5-25.45) | | 23.2 (21.325-25.825) | 0.62 | | |
| Energy intake (kcal/day)^a^ | | 1591 (1248-1945) | | 1875 (1548-2218) | 0.04^*^ | | |
| **Nutrients**  Animal protein  (% energy) ^a^ | | 7.64 (6.5575-9.82) | | 7.925 (6.8525-10.525) | 0.866 | | |
| Plant protein  (% energy) ^b^ | | 6.041 ± 1.2736 | 6.1483 ± 1.4128 | | 0.758 | |  |
| Animal fat  (% energy) ^b^ | | 13.576 ± 4.9613 | | 13.555 ± 4.1714 | 0.986 | | |
| Plant fat  (% energy) ^b^ | | 13.199 ± 4.2014 | 13.9873 ± 4.0447 | | 0.462 | |  |
| Carbohydrate  (% energy) ^b^ | | 49.0507 ± 9.0311 | 49.72 ± 8.0189 | | 0.763 | |  |
| Na (mg/kcal) ^a^ | | 2.22 (1.785-2.5775) | | 2.355 (2.0775-2.65) | 0.379 | | |
| K (mg/kcal) ^a^ | | 1.26 (1.0725-1.6425) | | 1.165 (1.0125-1.48) | 0.395 | | |
| Ca (μg/kcal) ^a^ | | 240.83 (201.14-443.94) | | 250.76 (190.97-326.43) | 0.572 | | |
| Mg (μg/kcal) ^a^ | | 123.49 (113.59-158.11) | | 126.65 (110.919-144.3) | 0.786 | | |
| Phosphorus (μg/kcal) ^a^ | | 536.82 (461.39-653.7) | | 546.69 (462.2-575.95) | 0.644 | | |
| Fe (μg/kcal) ^b^ | | 4.2587 ± 1.3843 | | 4.173 ± 1.03299 | 0.787 | | |
| Zn (μg/kcal) ^b^ | | 4.4783 ± 1.0591 | | 4.261 ± 0.6768 | 0.348 | | |
| Cu (μg/kcal) ^a^ | | 0.5967 ± 0.1447 | | 0.5857 ± 0.1088 | 0.741 | | |
| Mn (μg/kcal) ^a^ | | 1.375 (1.130-1.8375) | | 1.45 (1.285-1.8725) | 0.525 | | |
| Retinol (μg/kcal) ^a^ | | 0.19 (0.12-0.3675) | | 0.235 (0.1425-0.365) | 0.652 | | |
| β-carotene (μg/kcal) ^a^ | | 1.78 (0.9075-2.355) | | 1.27 (0.9575-2.355) | 0.626 | | |
| Vitamin A  (μg RAE/kcal) ^a,c^ | | 0.4 (0.2525-0.555) | | 0.385 (0.2725-0.4975) | 0.9 | | |
| Vitamin D (ng/kcal) ^a^ | | 4.315 (3.1325-7.8725) | | 4.74 (4.025-6.48) | 0.626 | | |
| α-tocopherol (μg/kcal)^b^ | | 3.932 ± 1.293 | 3.779 ± 0.969 | | 0.606 | |  |
| Vitamin K (μg/kcal)^a^ | | 0.165 (0.1125-0.23) | | 0.165 (0.095-0.2075) | 0.496 | | |
| Vitamin B1 (μg/kcal) ^b^ | | 0.423 ± 0.1319 | | 0.399 ± 0.0836 | 0.403 | | |
| Vitamin B2 (μg/kcal) ^b^ | | 0.7353 ± 0.2401 | | 0.7063 ± 0.1868 | 0.604 | | |
| Niacin (μg/kcal) ^a^ | | 8.76 (6.97-10.53) | | 8.765 (8.2425-10.4125) | 0.796 | | |
| Vitamin B6 (μg/kcal) ^a^ | | 0.65 (0.5525-0.8025) | | 0.615 (0.5525-0.73) | 0.734 | | |
| Vitamin B12 (ng/kcal)^a^ | | 3.735 (2.5825-5.48) | | 3.995 (3.255-5.04) | 0.264 | | |
| Foric acid (μg/kcal)^a^ | | 0.17 (0.12-0.23) | | 0.17 (0.1325-0.1975) | 0.847 | | |
| Vitamin C (μg/kcal) ^a^ | | 44.555 (32.475-69.518) | | 47.965 (34.77-61.848) | 1 | | |
| SFA (% energy) ^b^ | | 7.453 ± 2.5586 | | 7.6147 ± 2.0681 | 0.789 | | |
| MUFA (% energy) ^b^ | | 9.5143 ± 2.560 | 9.8533 ± 2.2387 | | 0.587 | |  |
| *n*-3PUFA (% energy) ^a^ | | 1.12 (0.9825-1.335) | 1.26 (1.035-1.4075) | | 0.304 | |  |
| *n*-6PUFA (% energy) ^b^ | | 5.1177 ± 1.3790 | | 5.3653 ± 1.3396 | 0.483 | | |
| Cholesterol (μg/kcal) ^a^ | | 193.39 (148.15-317.06) | 186.78 (136.51-276.85) | | 0.504 | |  |
| Alcohol (% energy) ^a^ | | 0.7 (0-13.3775) | | 3.92 (0-10.57) | 0.655 | | |
| **Foods** | |  | |  |  | | |
| Cereals (mg/kcal) ^a^ | | 174.24 (139.9-252.05) | | 187.54 (162.60-239.92) | 0.432 | | |
| Potatoes (mg/kcal) ^a^ | | 18.11 (11.22-26.72) | 12.07 (8.09-18.71) | | 0.109 | |  |
| Pulses (mg/kcal) ^a^ | | 2.002 (1.106-2.815) | | 1.704 (0.741-2.338) | 0.219 | | |
| Green and yellow vegetables (mg/kcal) ^a^ | | 55.767 (28.102-68.577) | | 57.522 (31.895-71.154) | 0.797 | | |
| Other vegetables (mg/kcal)^a^ | | 73.553 (51.690-99.447) | | 56.374 (39.669-81.401) | 0.0878 | | |
| Fruit (mg/kcal) ^a^ | | 40.917 (12.955-61.66) | | 50.238 (31.728-81.382) | 0.254 | | |
| Fish and shellfish (mg/kcal) ^a^ | | 26.017 (17.661-42.356) | | 28.848 (21.682-33.563) | 0.564 | | |
| Meat (mg/kcal) ^a^ | | 44.33 (26.84-55.63) | | 44.68 (28.98-57.52) | 0.719 | | |
| Eggs (mg/kcal)^a^ | | 20.716 (12.993-34.575) | | 17.207 (12.922-29.557) | 0.708 | | |
| Dairy products (mg/kcal) ^a^ | | 71.505 (31.427-102.256) | | 73.97 (16.293-102.245) | 0.631 | | |
| Oils and fats (mg/kcal)^b^ | | 5.8495 ± 2.8658 | | 6.6083 ± 2.9869 | 0.32 | | |
| Confection (mg/kcal)^a^ | | 19.1402 (10.502-26.606) | 13.234 (7.931-32.505) | | 0.304 | |  |
| Beverages (mg/kcal)^a^ | | 380.88 (261.55-465.21) | | 392.99 (280.66-628.40) | 0.398 | | |
| Seasonings and spices (mg/kcal)^a^ | | 104.02 (87.23-131.80) | | 136.78 (98.54-184.14) | 0.0591 | | |
| Sugar and sweeteners (mg/kcal)^a^ | | 2.002 (1.106-2.8149) | | 1.704 (0.7413-2.3381) | 0.219 | | |

^a^ Data provided as median (interquartile range), analyzed by Mann–Whitney U-test.

^b^Data provided as mean ± standard deviation, analyzed by Student’s *t*-test.

^c^ Vitamin A (μg RAE/kcal) is equal to retinol (μg/kcal) + 1/12 × β-carotene (μg/kcal) +1/24 × α-carotene (μg/kcal) + 1/24 × β-cryptoxanthin (μg/kcal) +1/24 × other carotenoids (μg/kcal).

^d^ Fisher’s exact test was used to test the significance of differences in frequency distribution.

^*^ Significant differences at *p* < 0.05, ^**^*p* < 0.01.

SFA, saturated fatty acid; MUFA, monounsaturated fatty acid; PUFA, polyunsaturated fatty acid; RAE, retinoic acid equivalent

| Supplemental table 2. The association of moderate-to-severe vitiligo with each variable tested by multivariate logistic regression analysis | | | |
| --- | --- | --- | --- |
|  | Odds ratio | 95% confidential interval | *p* |
| (Intercept) | 0.421 | 0.00374 - 47.3 | 0.719 |
| Age | 0.975 | 0.927 - 1.03 | 0.323 |
| Sex [T.F] | 0.106 | 0.0263 - 0.425 | 0.00155** |
| Body mass index | 1.02 | 0.873 - 1.2 | 0.771 |
| Disease duration | 1.1 | 1 -1.22 | 0.0437* |
| Energy intake | 1 | 1 - 1 | 0.078 |
| *Statistically significant at *p* < 0.05, ** at *p* < 0.01. | | | |
